# A novel simplified structural design as an artificial enzyme for efficient hydrolysis of PNPA

**DOI:** 10.1038/s41598-025-92439-1

**Published:** 2025-03-17

**Authors:** Wenfang Li, Yuze Lu, Jiajun Wang, Chuanbi Li

**Affiliations:** Key Laboratory of Preparation and Application of Environmentally Friendly Materials, Ministry of Education, College of Chemistry, Jilin Normal University, Changchun, 130103 China

**Keywords:** Biochemistry, Chemical biology

## Abstract

In this study, a novel artificial enzyme Zn(II)-SMM with simple organic structure for PNPA-directed hydrolysis is proposed. The introduction of hydrophobic quinoline and dipyridinium in Zn(II)-SMM system demonstrates high activity for the complexation of Zn^2+^ and hydrolysis of PNPA, and the catalytic rate (k_cat_/k_uncat_) exceeds 5239 times that of non-catalytic systems. The newly designed small molecule organic complex effectively binds to and catalyzes the hydrolysis of substrate PNPA in accordance with Michaelis-Menten kinetics. The mechanism with activation by single Lewis acid provides fundamental insights for the development of small molecule hydrolases.

**Supplementary Information:**

The online version contains supplementary material available at 10.1038/s41598-025-92439-1.

Artificial enzymes have been developed in the last two decades due to their unique catalytic performances and potential applications^1,2^. By emulating the catalytic mechanisms of natural enzymes, these synthetic catalysts exhibit specific substrate recognition and efficient conversion capabilities. The selectivity and catalytic activity can be enhanced through structural modifications, and this has resulted in several noteworthy advancements, including enzyme mimics derived from supramolecular scaffolds^3–6^, molecular imprinting^7–9^, dendritic macromolecules^10,11^, small molecules^12,13^, nanomaterials^14–17^ and so on^18,19^. In summary, the diversity and abundance of artificial enzymes are vast. For example, in the hydrolysis of phosphate esters, the design of multi-nuclear catalytic sites is typically a prerequisite for achieving highly efficient catalysts. Consequently, binuclear metal complexes^20^, binuclear zinc(II) complex with hydrogen bond donors^21^, the asymmetric binuclear ligand^22^, and complexes with FeIII-FeIII and (FeIII-FeII) configurations^23^ etc. have been developed. These designs aim to achieve catalytic efficiencies comparable to those of natural phosphatases. However, it is evident that the structure of phosphatases is relatively rigid and their application scope is limited. In contrast, the design of artificial enzymes allows optimization of both catalytic center and substrate binding site structures, offering greater flexibility in design. This versatility enables application in a wide range of chemical reactions and biological processes, thereby providing expanded opportunities for catalyst development.

Compared to other types of enzymes^24–28^, small molecular enzymes demonstrate superior stability and ease of preparation relative to their natural counterparts. Therefore, the development of small.

molecule-based metallohydrolase (SMM) with high catalytic ability has received considerable attention.A particularly critical factor in this process is the incorporation of metal ions such as zinc, iron, copper, etc., which confers unique catalytic properties and selectivity.

In this article, we present a novel artificial enzyme constructed by simple organic structure designed for the directed hydrolysis of esters. Hydrophobic quinoline was incorporated to mimic the flexible action of natural hydrolases and enhance the binding affinity between the enzyme and the substrate. Additionally, dipyridinium was strategically positioned as a binding site due to its strong capacity for metal ion coordination, and Zn^2+^ ions coordinated by nitrogen atoms in tertiary amines, pyridine, quinolone, and acyl-oxygen bonds was introduced to function as the catalytic metal center (as illustrated in Fig. 1). Consequently, a small molecular enzyme complex Zn(II)-SMM is obtained.

**Fig. 1:**
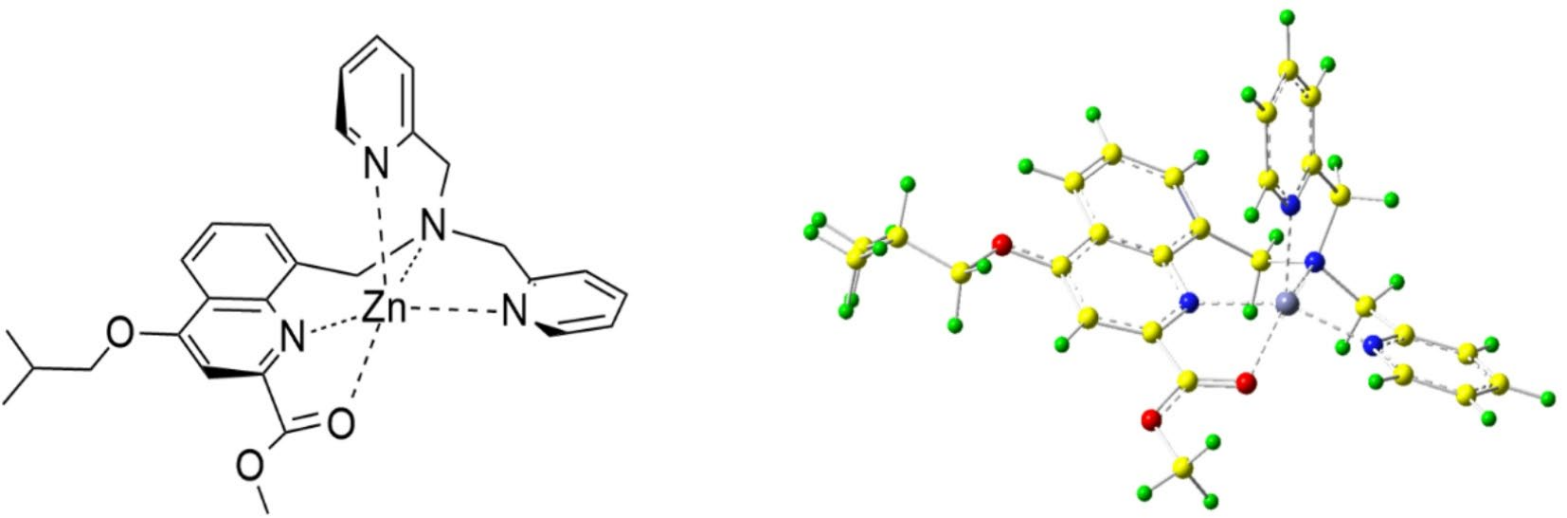
The chemical structure (left) and the simulated model (right) of the Zn(II)-SMM complex.

The chemical structure is created by ChemDraw software and the theoretical calculations are carried out using Gaussian 16 A.03 package under b3lyp/6–31 g(d, p) level. The calculations involved both vacuum and conductor-like polarizable continuum model. The presented geometries are optimized structures under vacuum and water circumstances of the Zn^2+^ coordinate.

## Results

### Eevaluation of catalytic efficacy of Zn(II)-SMM

 p-nitrophenyl acetate (PNPA) was selected as a typical substrate for the evaluation of catalytic efficacy of Zn(II)-SMM. Notably, the slope of reaction rates increased significantly with rising concentrations of PNPA from 20 µM to 100 µM compared to spontaneous reactions under identical conditionsFig. . 2b and Figure S3), indicating that Zn(II)-SMM exhibited unexpectedly high activity for hydrolysis of PNPA hydrolysis in mixtures of HEPES-DMSO and an incremental rate of hydrolysis.

**Fig. 2:**
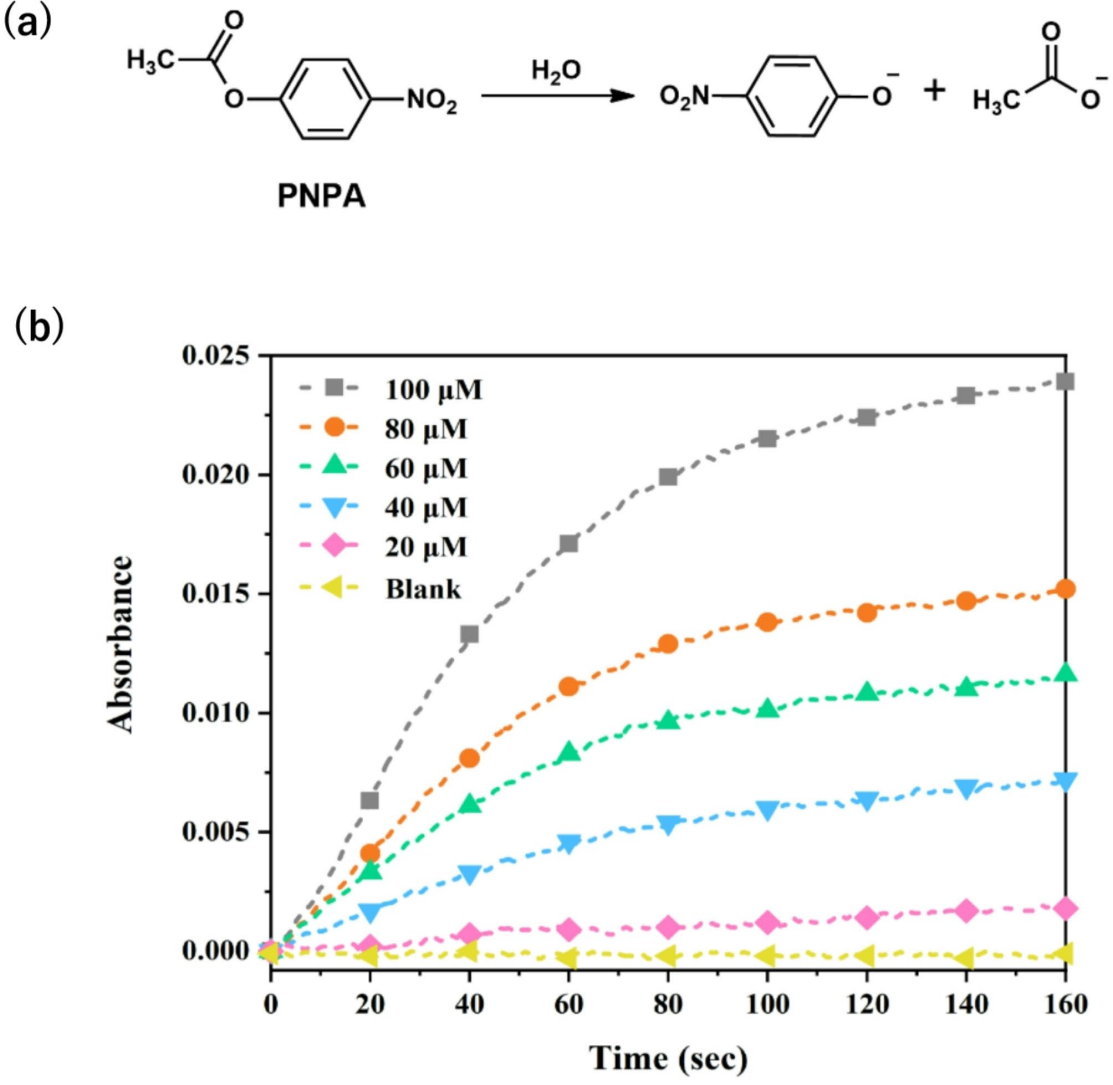
(**a**) The hydrolysis reaction for the substrate p-nitrophenyl acetate (PNPA). (**b**) Plots of absorbance vs. time at 400 nm in HEPES of different concentrations for substrate PNPA assisted by 10 µM Zn(II)-SMM in a DMSO/ H EPES mixture (20 : 80 v/v) at 25 ◦C and pH 7.0, Blank: DMSO / HEPES mixture (20 : 80 v/v) at 25 ◦C and pH 7.0 .

**Fig. 3:**
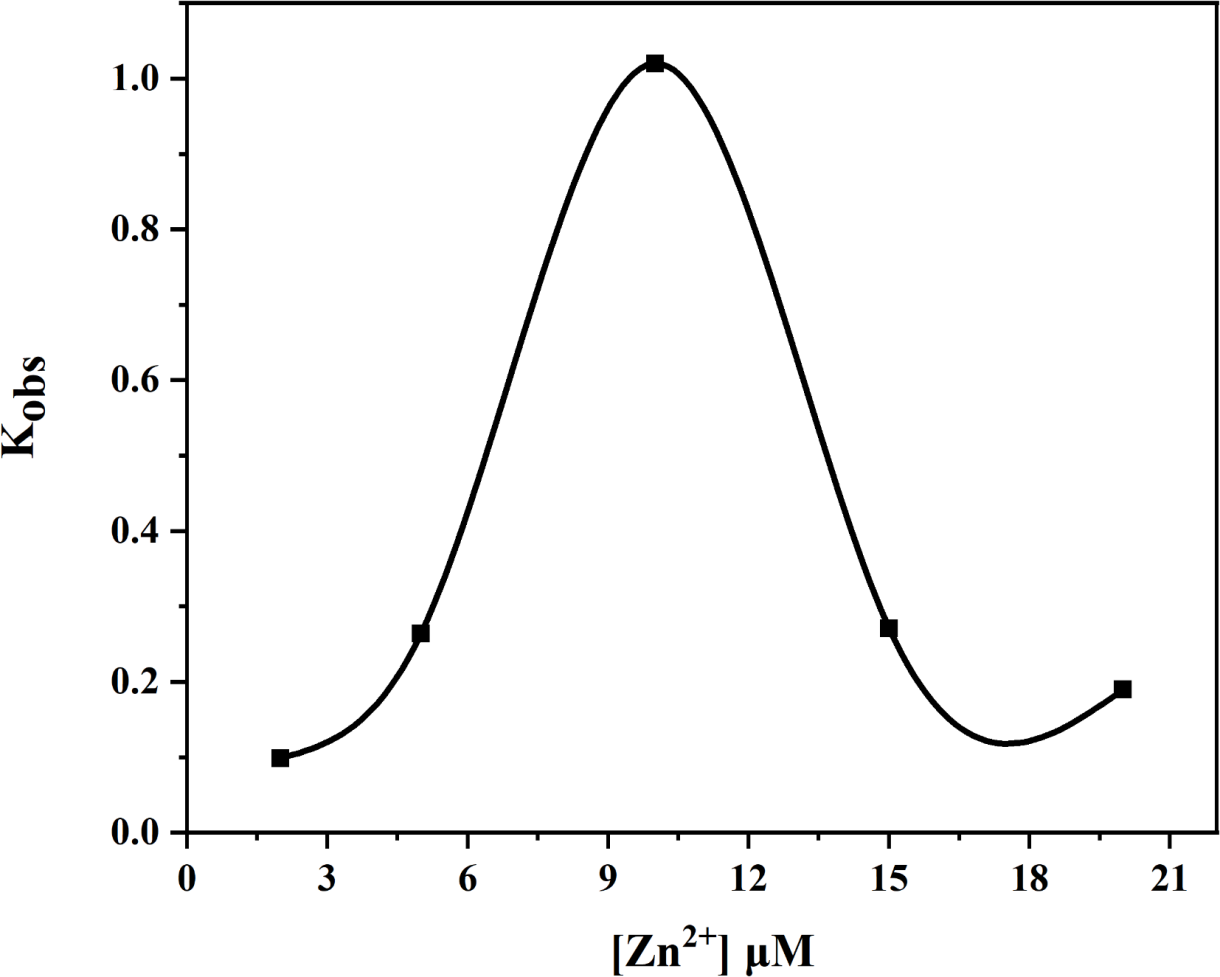
Plots of K_obs_ for the hydrolysis of PNPA (50 µM) by Zn(II)-SMM (10 µM) in DMSO/HEPES mixture (20 : 80 v/v) with the increase concentration of Zn^2+^ at 25 ◦C and pH 7.0.

Even upon introducing tenfold excess substrate amounts, catalytic hydrolysis consistently concluded within approximately sixty seconds—highlighting the remarkably high catalytic efficiency of this enzyme system (Fig. 2). The apparent rate constant K_obs_ was determined by varying concentrations of Zn^2+^ (Fig. 3), K_obs_ gradually increased until reaching a molar ratio of Zn^2+^ to SMM at 1:1—suggesting that the hydrolase complex Zn(II)-SMM comprised one mole each of Zn^2+^ and SMM.

### Computational insights

To elucidate the binding mechanism of SMM with Zn^2+^, energy simulation calculations were conducted. The energy simulations reveal that Zn^2+^ adopts a characteristic planar structure involving acyl oxygen and nitrogen atoms, achieving maximum stability in a tetrahedral configuration with the nitrogen atom on pyridine Fig.  1). This configuration is energetically favored by 99.62 kJ/mol compared to alkyl-oxygen coordination (Figure S2), utilizing the b3lyp/6–31 g(d, p) method via Gaussian software.

### UV-vis monitoring for catalytic effects

The primary architecture of the hydrolase was engineered using a quinoline derivative, facilitating the proximity of PNPA with analogous organic structure and interaction with metal ions. In this study, the hydrolysis of PNPA yielded phenol, which exhibited UV absorption at 400 nm^29^. To gain deeper insights into the catalytic effect, UV monitoring experiments were conducted (Figure S1). The results indicated that phenol concentration increased over time; however, no hydrolysis occurred with the removal of SMM (Figure S3), implying that Zn(II)-SMM could associate with the substrate and promote the hydrolysis of it.

### Kinetics

The kinetics of catalytic hydrolysis of Zn(II)-SMM on PNPA were investigated in detail to elucidate the behavior of the catalytic hydrolysis. The experimental findings showed a characteristic double reciprocal plot and saturation kinetics curve (Figure S4), confirming that the catalytic activity adheres to the Michaelis-Menten Eq. 30, thereby indicating enzyme-like characteristics of the Zn(II)-SMM complex. The calculated kinetic parameter value was 0.8287 min^-1^ (K_cat_), and the hydrolysis rate is 5239 times greater than that of the spontaneous reaction.

### Mechanism and pathway

The hydrolysis rate constant of Zn(II)-SMM system was evaluated across various pH levels, and the results (Figure S5) indicated that following the activation of Lewis acid within the system, the monohydroxyl form of the metal ion complex acted as an effective catalyst for.

**Fig. 4:**
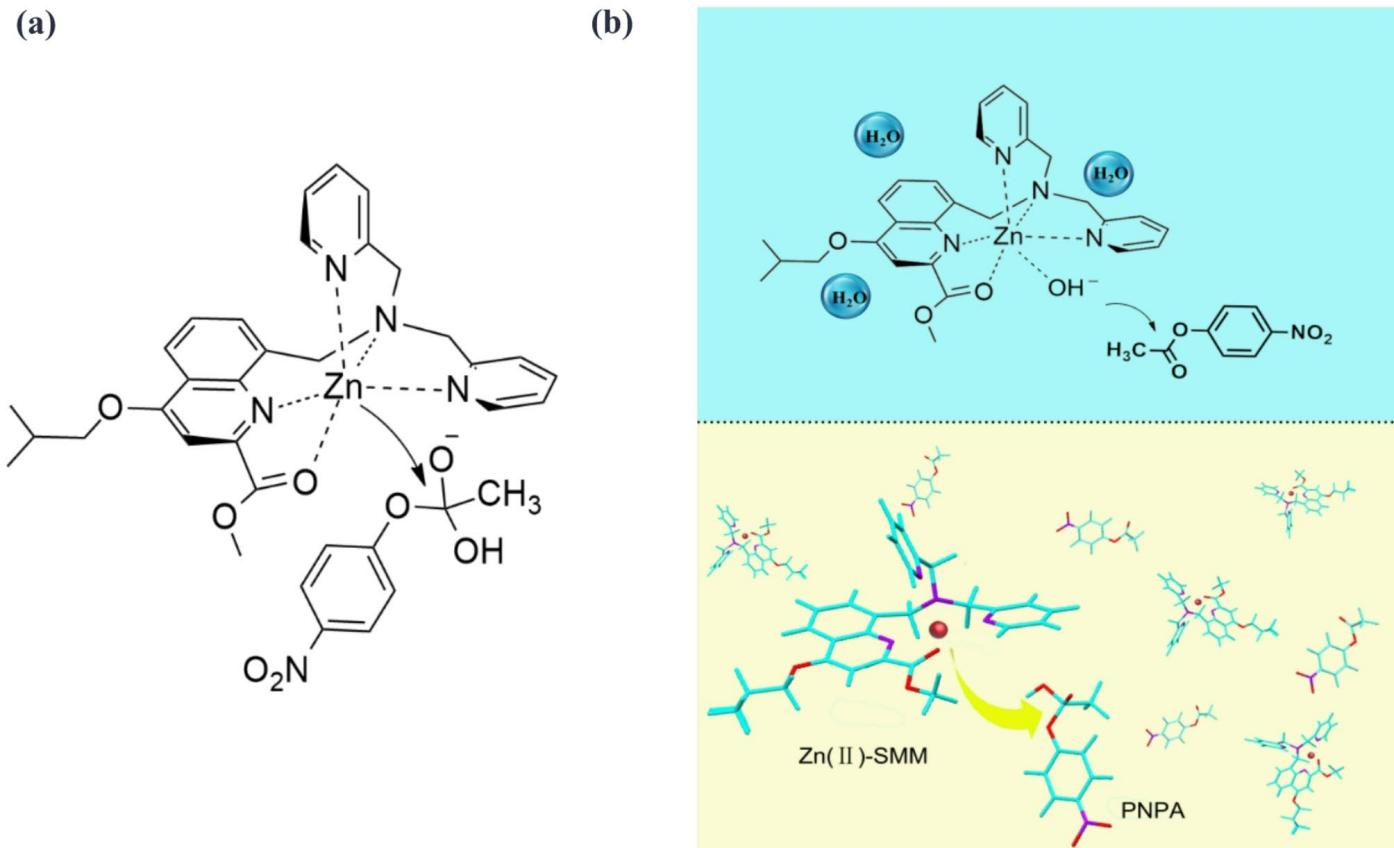
(**a**) Mechanism of PNPA hydrolysis catalyzed by SMM. (**b**) Schematic representation of the hydrolysis mechanism for PNPA.

PNPA hydrolysis. Under acidic conditions, the pyridine moiety of the Zn(II)-SMM complex readily.

associates with H^+^, hindering substrate access and resulting in infrequent hydrolysis events. Conversely, as pH increased, deprotonation of the Zn(II)-SMM complex allowed water to coordinate with Zn^2+^,

forming a Zn-OH bond that facilitates nucleophilic attack on PNPA, thereby accelerating its hydrolysis.

The proposed hydrolysis mechanism of PNPA under neutral conditions is also illustrated in Fig. 4. Here, the catalytic site of Zn-OH functions as either a general base or nucleophile to activate the C-O carbonyl bond and generate a transition state conducive to cleavage of this bond and subsequent release of p-nitrophenol, the mechanism is hypothesized to align with that of single Lewis acid activation^31–33^.

## Discussion

A novel small molecular organic complex capable of coordinating with Zn^2+^ has been designed, which effectively binds and catalyzes the hydrolysis of the substrate PNPA. This system has been proved to own the ability to mimic the catalytic hydrolysis function of metal hydrolases, and the catalytic rate (k_cat_/k_uncat_) exceeds 5239 times that of non-catalytic systems. Furthermore, saturation kinetics studies indicate that the catalytic process of the Zn(II)-SMM complex adheres to the Michaelis-Menten equation, identical to natural enzymes. The hydrolysis mechanism employed by this engineered metal hydrolase is characterized as a single Lewis acid activation. The results of this work elucidates that the artificial metal hydrolase Zn(II)-SMM system is a promising candidate for the design of small molecule hydrolases, and it is believed that this facile route to construct metal hydrolases provides a convenient path for catalytic research, most importantly, it lays the foundation for the further pursuit of our research on chiral metallohydrolase.

## Methods

### Materials and physical measurements

All chemicals including o-toluidine, bis (pyridin-2-ylmethyl) amine etc. and solvents were of pure analytical grade and obtained commercially from aladdin company. The synthesized small molecule-based metallohydrolase (SMM) prepared via organic synthesis method were characterized through 1 H NMR, 13 C NMR, and MALDI-TOF-MS.


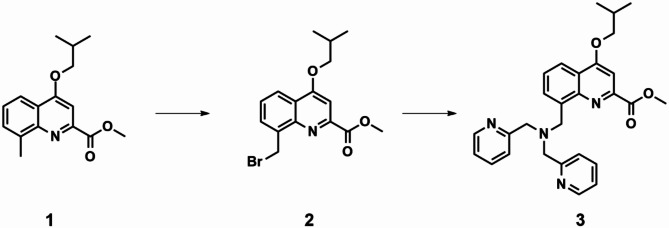


### Synthesis of SMM compound and characterizations

#### Synthesis of compound 2

Compound 1(10 g, 36.6 mmol)), NBS (6.83 g, 38.4 mmol, 1.05 eq) and BPO (9.3 g, 38.4 mmol, 1.05 eq) were dissolved in 100 mL of benzene at 80 °C. The mixture was refluxed for 6 h. the organic solvent was then removed under reduced pressure, the residue was washed with dichloromethane and water. The product was purified by column chromatography with 200–300 mesh silica gel column. The collected product was dried under reduced pressure to give a white solid (9.8 g, 76% yield).

#### Synthesis of compound 3

Compound 2 (700 mg, 2.06 mmol), 2,2’ -dimethylpyridinolamine (0.82 g, 4.12 mmol), potassium carbonate (1.13 g, 8.23 mmol), Anhydrous DMF(10 mL) was mixed and stirred for 1.5 h, then diluted with 1 M HCl, and washed with AcOEt, the organic phase was dried with MgSO4, and the solvent was removed in vacuum, the target compound 3 (773 mg, 65% yield) was purified by column chromatography ( silica gel, CHCl3/ MeOH ).

## Electronic supplementary material

Below is the link to the electronic supplementary material.

Supplementary Material 1

## Data Availability

All data generated or analysed during this study are included in this published article and its supplementary information files.
