# Supplementary material for: A novel simplified structural design as an artificial enzyme for efficient hydrolysis of PNPA
Source: Sci Rep. 2025 Mar 17;15:9071. doi: 10.1038/s41598-025-92439-1 (PMC11914156; doi:10.1038/s41598-025-92439-1)
Supplement: Supplementary file 1 — Supplementary Material 1 [file 41598_2025_92439_MOESM1_ESM.docx]

**Efficient hydrolysis of PNPA utilizing artificial enzyme with simple structure: Kinetics, mechanism and pathway**

Wenfang Li*, Yuze Lu, Jiajun Wang & Chuanbi Li

**Table of Contents**

[Figure S1. Absorbance plot of substrate](#_Toc177974707) PNPA……………………………………...S2

[Figure S2. simulated structure of the Zn(Ⅱ)-SMM complex coordinating with Zn^2+^S2](#_Toc177974708)

[Figure S3. Comparison of Zn(Ⅱ)-SMM complex catalyzing hydrolysis of PNPA with and without SMM…………………………………………………………………....S3](#_Toc177974709)

[Figure S4. Double-reciprocal plots and diagram of saturation kinetics for the catalysis of Zn(Ⅱ)-SMM complex (10 μM)………………………………….……….............S4](#_Toc177974710)

[Figure S5. Dependence of K_obs_ on increasing pH value for hydrolysis of PNPA…...S4](#_Toc177974711)

[Figure S6 The synthetic procedure of SMM…………………………………………S5](#_Toc177974712)

[Compound Characterizations………………………………………………………...S5](#_Toc177974713)

[NMR data…………………………………………………………………………….S6](#_Toc177974714)

Mass Spectrum……………………………………………………………………….S6

Key Laboratory of Preparation and Application of Environmentally Friendly Materials, Ministry of Education, College of Chemistry, Jilin Normal University, Changchun, 130103, China. *email: liwf12@jlnu.edu.cn.


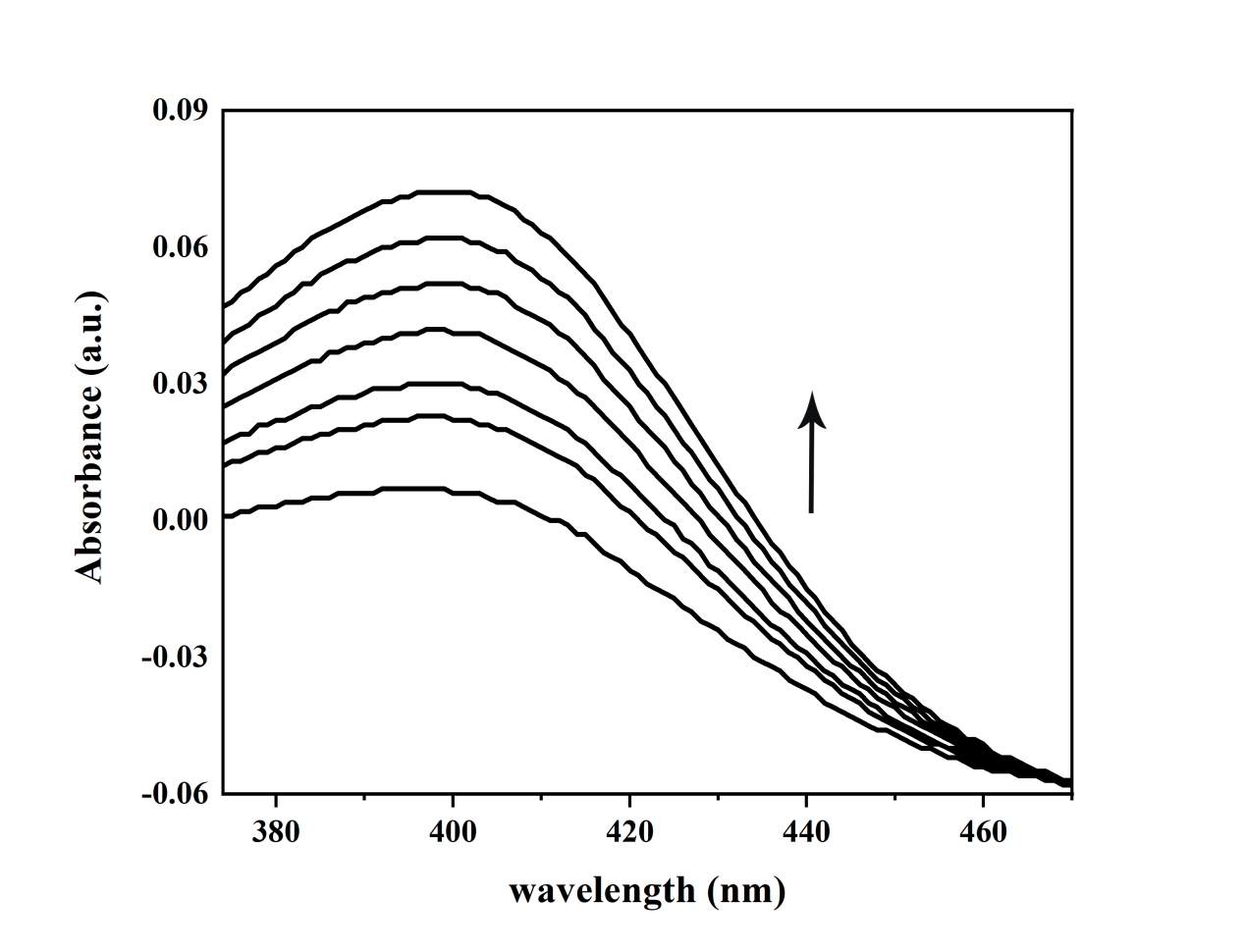
Figure S1. Absorbance plot of substrate PNPA (100 μM) measured in 9 minutes with every 1.5 minute intervals in the presence of Zn(Ⅱ)-SMM (10 μM), Zn^2+^ (20 μM) in a DMSO/HEPES mixture (20 : 80 v/v) at 25 ℃ and pH 7.0.


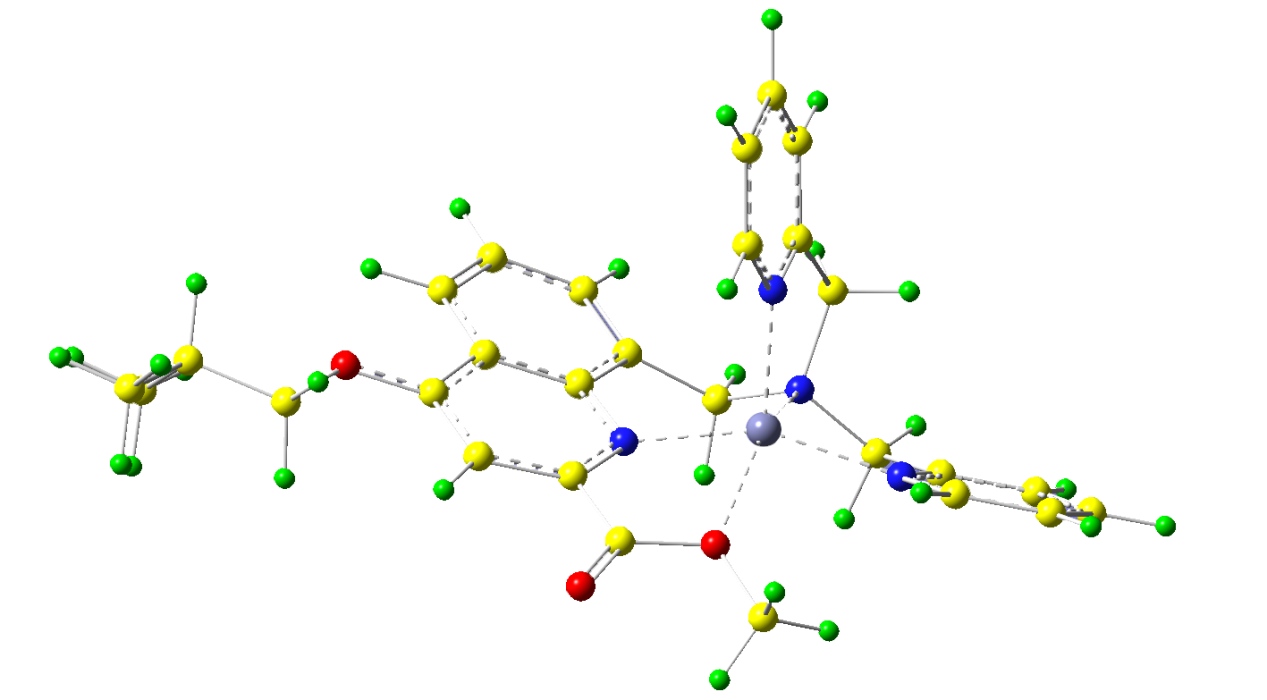


# Figure S2. simulated structure of the Zn(Ⅱ)-SMM complex coordinating with Zn^2+^ by alkyl-oxygen.


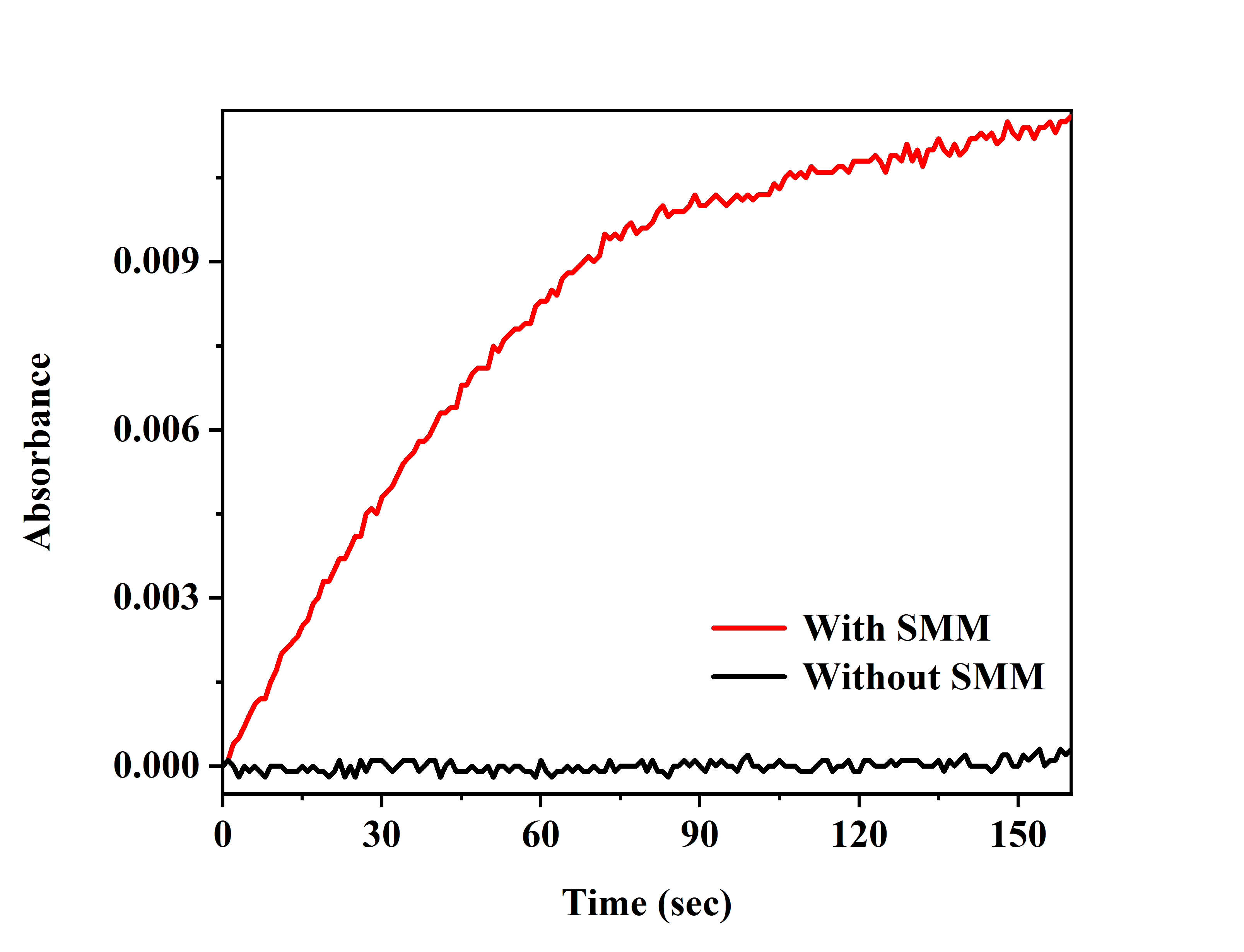


# Figure S3. Comparison of Zn(Ⅱ)-SMM complex catalyzing hydrolysis of PNPA with and without SMM under same conditions: 10 μM SMM, 10 μM Zn^2+^, 40 μM PNPA in DMSO / HEPES mixture (20 : 80 v/v) at 25 ◦C and pH 7.0.


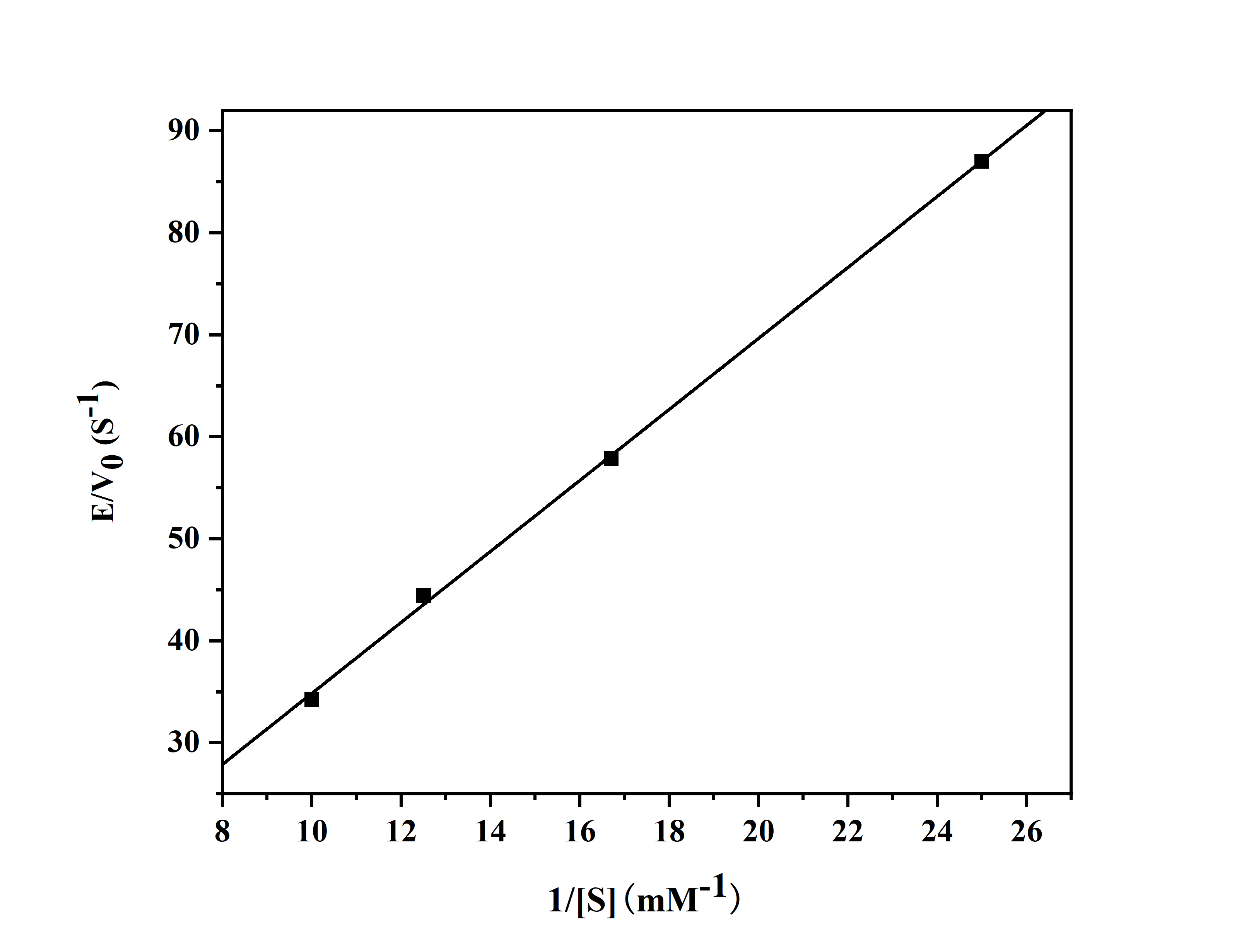


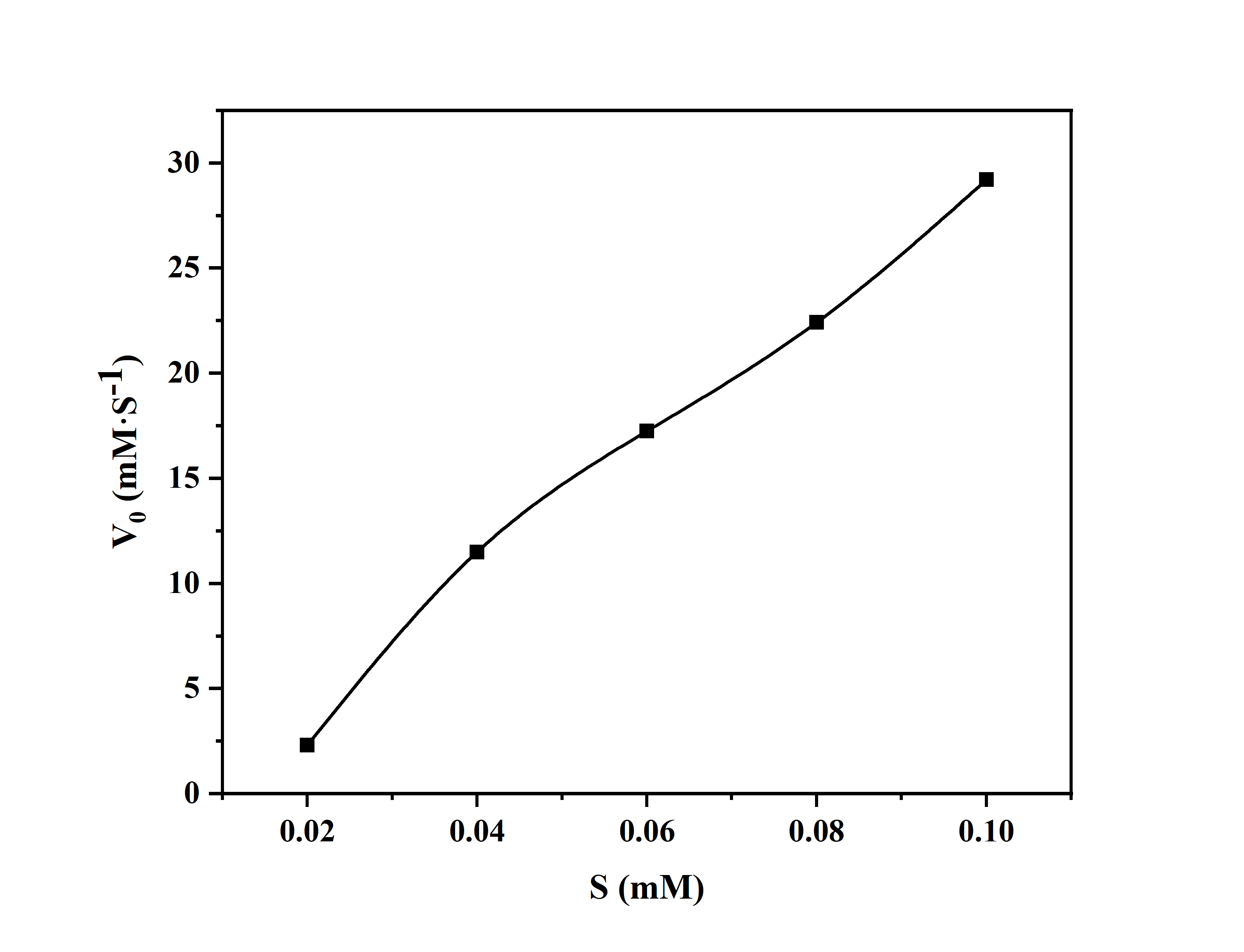


**(b)**

# Figure S4. (a) Double-reciprocal plots and (b) diagram of saturation kinetics for the catalysis of Zn(Ⅱ)-SMM complex (10 μM) in DMSO/HEPES (20:80, v/v) mixture at 25 °C and pH 7.0.


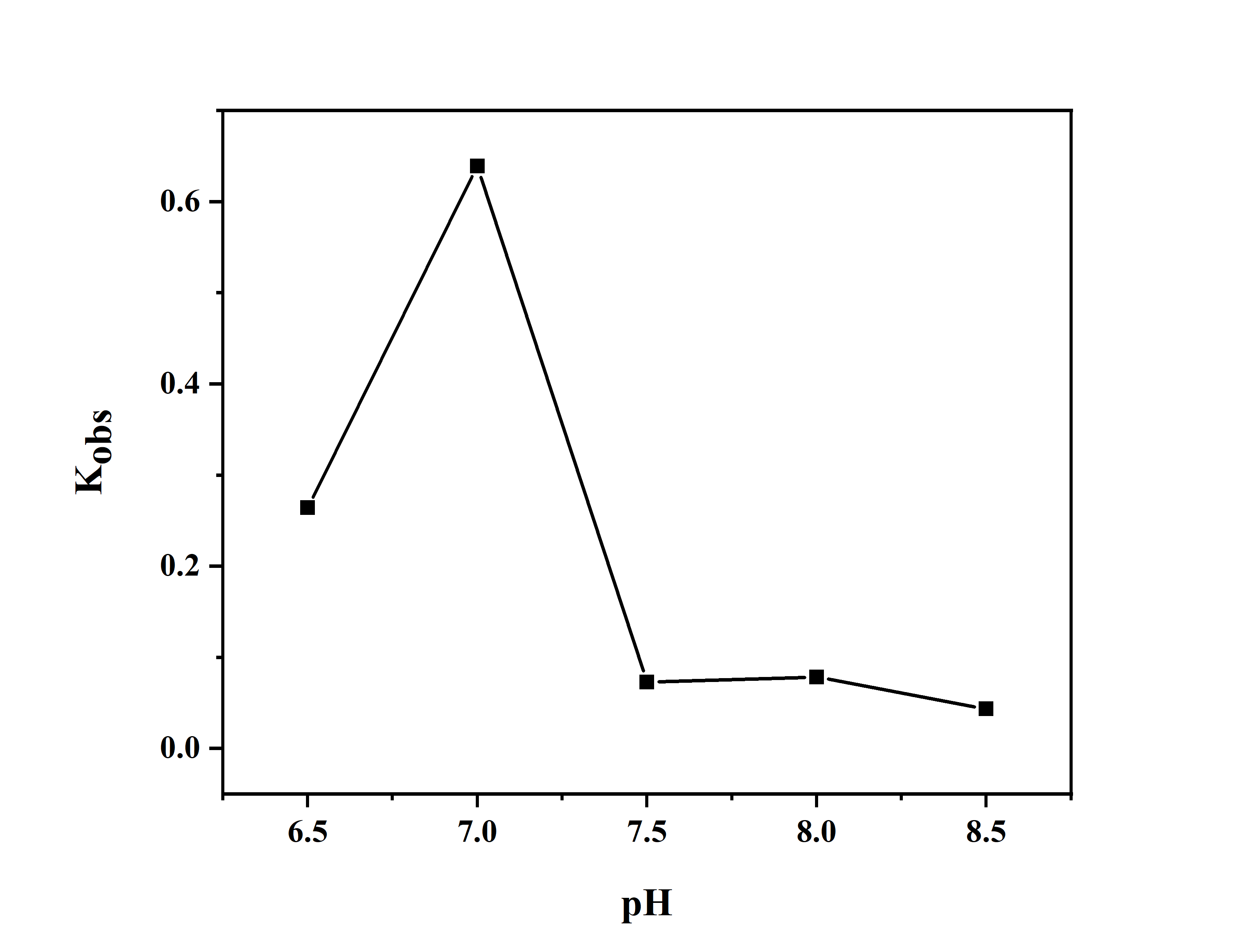


# Figure S5. Dependence of K_obs_ on increasing PH value for hydrolysis of PNPA.


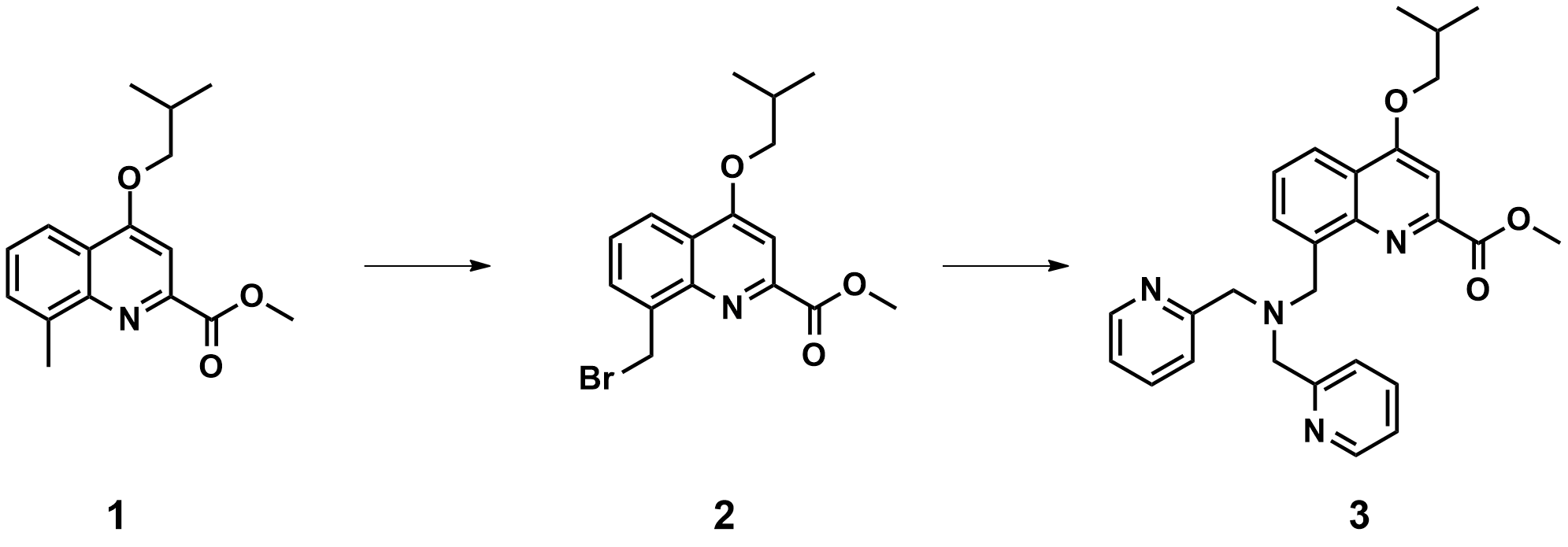


# Figure S6. The synthetic procedure of the small molecule-based metallohydrolase (SMM).

# **Compound Characterizations**

Chemical characterization of compound **2**

^1^H NMR (500 MHz, CDCl_3_) δ 8.23 (d, *J* = 8.4 Hz, 1H), 7.90 (d, *J* = 7.1 Hz, 1H), 7.57 – 7.53 (m, 2H), 5.31 (s, 2H), 4.08 – 4.01 (m, 5H), 2.28 (dp, *J* = 13.3, 6.7 Hz, 1H), 1.13 (d, *J* = 6.7 Hz, 6H). ^13^C NMR (126 MHz, CDCl_3_) δ 166.39, 162.84, 148.69, 146.02, 136.96, 131.67, 127.12, 122.52, 101.01, 77.25, 77.00, 76.75, 75.11, 53.02, 29.40, 28.16, 19.21. MS (TOF MS ES+): calcd for [C16H18BrNO3+H]^+^: 353.22; found: 353.32.

Chemical characterization of compound **3**

^1^H NMR (500 MHz, CDCl_3_ ) δ 8.49 (d, *J* = 4.2 Hz, 2H), 8.15 (dd, *J* = 16.5, 7.6 Hz, 2H), 7.77 (t, *J* = 7.6 Hz, 2H), 7.74 – 7.66 (m, 3H), 7.54 (s, 1H), 7.27 – 7.22 (m, 2H), 4.37 (s, 2H), 4.12 (d, *J* = 6.3 Hz, 2H), 3.95 (s, 3H), 3.85 (s, 4H), 2.21 (dt, *J* = 12.8, 6.5 Hz, 1H), 1.09 (d, *J* = 6.7 Hz, 6H). ^13^C NMR (500 MHz, CDCl_3_) δ = 166.55, 162.75, 160.16, 149.02, 147.99, 147.19, 138.17, 136.45, 129.69, 127.28, 122.45, 122.30, 121.81, 120.52, 100.58, 77.37, 77.12, 76.87, 74.95, 60.48, 53.55, 52.98, 28.19, 19.27. MS (TOF MS ES+): calcd for [C28H30N4O3+H]^+^:471.56; found: 471.11; [C28H30N4O3+Na]^+^: 493.82.

#

# NMR data


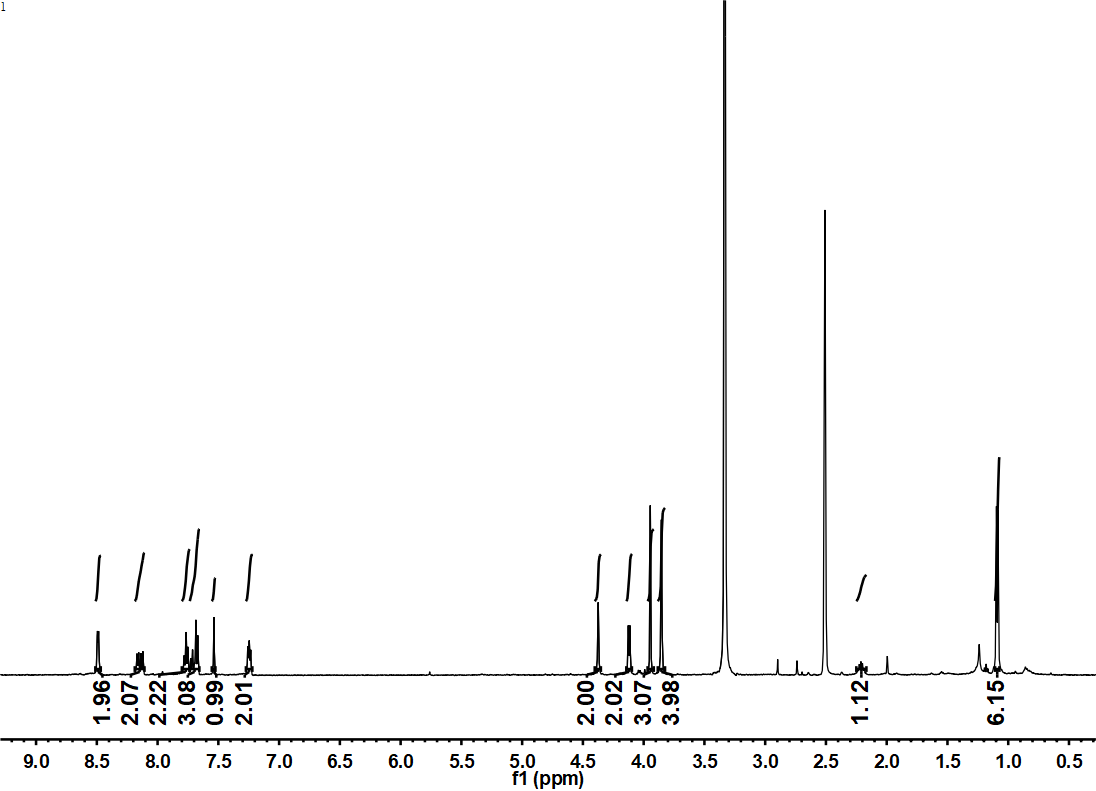

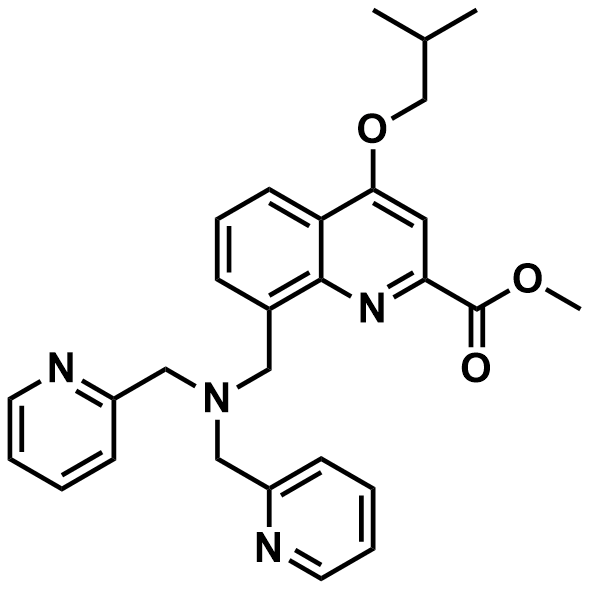

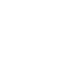


# Mass Spectrum


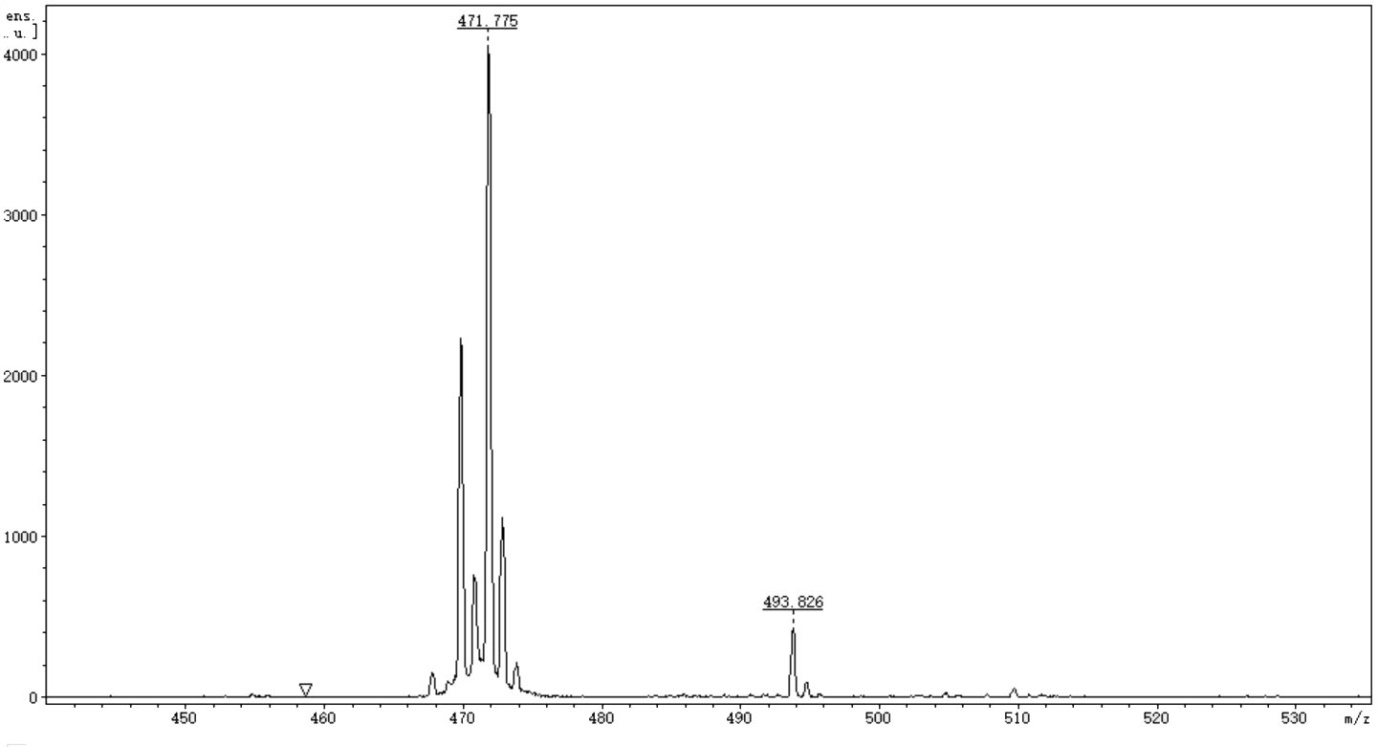


**SMM + Na^+^**

**SMM + H^+^**
